# Circulating microRNAs are deregulated in overweight/obese children: preliminary results of the I.Family study

**DOI:** 10.1186/s12263-016-0525-3

**Published:** 2016-03-21

**Authors:** Giuseppe Iacomino, Paola Russo, Ilaria Stillitano, Fabio Lauria, Pasquale Marena, Wolfgang Ahrens, Pasquale De Luca, Alfonso Siani

**Affiliations:** 1 Institute of Food Sciences, CNR, Via Roma, 64, 83100 Avellino, Italy; 2 Leibniz Institute for Prevention Research and Epidemiology - BIPS, Bremen, Germany; 3 Institute of Statistics, Faculty of Mathematics and Computer Science, University Bremen, Bremen, Germany; 4 BioGeM, Ariano Irpino, Italy

**Keywords:** Circulating miRNAs, Childhood obesity, Metabolic disorders, Biomarker

## Abstract

**Background:**

MicroRNAs (miRNAs) are small non-coding RNAs involved in the modulation of gene expression and in the control of numerous cell functions. Alterations of miRNA patterns frequently occur in cancer and metabolic disorders, including obesity. Recent studies showed remarkable stability of miRNAs in both plasma and serum making them suitable as potential circulating biomarkers for a variety of diseases and conditions.

The aim of this study was to assess the profile of circulating miRNAs expressed in plasma samples of overweight or obese (OW/Ob) and normal weight (NW) prepubertal children from a European cohort (www.ifamilystudy.eu). The project, aimed to assess the determinants of eating behavior in children and adolescents of eight European countries, is built on the IDEFICS cohort (www.ideficsstudy.eu), established in 2006. Among the participants of the I.Family Italian Cohort, ten OW/Ob (age 10.7 ± 1.5 years, BMI 31.6 ± 4.3 kg/m^2^) and ten NW (age 10.5 ± 2.7 years, BMI 16.4 ± 1.7 kg/m^2^) children were selected for the study. Gene arrays were employed to differentially screen the expression of 372 miRNAs in pooled plasma samples. Deregulated miRNAs (*p* < 0.05) were further validated in the individual samples using a real-time PCR (RT-qPCR) approach.

**Results:**

Using a significance threshold of *p* < 0.05 and a fold-change threshold of ± 4.0, we preliminarily identified in the pooled samples eight miRNAs that differed between the OW/Ob and NW groups. The validation by RT-qPCR in the individual plasma samples showed a twofold upregulation of miR-31-5p, a threefold upregulation of miR-2355-5p, and a 0.5-fold downregulation of miR-206 in OW/Ob as compared with NW. The molecular functions of these differentially expressed plasma miRNAs as well as their expected mRNA targets were predicted by bioinformatics tools.

**Conclusions:**

This pilot study shows that three circulating miRNAs are differentially regulated in OW/Ob as compared with NW children. Although causal pathways cannot be firmly inferred by these results, that deserve confirmation in larger samples, it is conceivable that circulating miRNAs may be novel biomarkers of obesity and related metabolic disturbances.

**Electronic supplementary material:**

The online version of this article (doi:10.1186/s12263-016-0525-3) contains supplementary material, which is available to authorized users.

## Background

The obesity epidemic represents a major health challenge worldwide since it is associated with severe adverse consequences for human health [1–3]. Excess energy intake and lack of physical activity are the main factors that drive the development of obesity. Individual traits, such as the genetic profile, likewise contribute to obesity. A quantity of studies has found a number of genes associated with obesity and obesity-related phenotypes [4–6].

Adipose tissue, the storage site of triglycerides, acts as an endocrine organ contributing to regulate energy homeostasis [7, 8]. Anomalous fat accumulation in obesity increases the risk of severe diseases such as metabolic syndrome, type 2 diabetes, atherosclerosis, and cancer [9]. Evidence suggests that in obesity, adipose tissues stay in a state of subclinical chronic inflammation [10] mainly prompted by the massive recruitment of macrophages into adipose tissues [11]. Adipogenesis is tightly controlled by a mixture regulatory signals including endocellular transcription factors and circulating hormones [12]. Additional regulators of adipogenesis also include microRNAs (miRNAs) [13–17]. These small non-coding RNAs, which have a length of only 20–24 nucleotides, are involved in the modulation of gene expression and, consequently, in the control of numerous cell functions [18–20]. At present, more than 2.000 different miRNAs have been described in humans and their number is still increasing (miRBase) (http://www.mirbase.org). miRNAs are important elements of the cell epigenetic machinery that, opposing to the other known players of epigenetic regulation (DNA methylation and post-translational histone modifications), can affect gene expression by binding to the 3′ untranslated sequence of a target messenger RNA (mRNA) [21]. Each miRNA can target many transcripts, and transcription levels of a gene may be regulated by multiple miRNAs [21, 22]. Computational and experimental analyses indicate that endogenous miRNAs regulate the expression of up to 30 % of mouse and human genes [23]. miRNAs have been shown to be involved in fundamental cellular processes including proliferation, differentiation, DNA repair, apoptosis, and metabolism [24, 25].

As a consequence, remarkable evidence indicates that dysregulation of miRNAs is causative and/or indicator of a number of disease processes including cancer [26]. Altered circulating miRNA profiles have been linked to numerous cardiometabolic diseases, including hypertension (let-7e), type 2 diabetes (miR-126), hepatic injury (miR-122), and atherosclerosis (miR-223) [14].

The presence of extracellular circulating miRNAs in blood and other biological fluids is well established [27, 28], but the reason for the nuclease resistance of miRNAs outside the cell remained unclear for a long period [29]. The discovery that cell-free miRNAs are detectable in serum and plasma and that their expression varies as a result of disease offers great potential for the discovery of novel health/disease biomarkers and candidate therapeutic targets [30].

Various miRNAs have been recognized to play a role in adipogenesis [31]. Preliminary evidence showed that some circulating miRNAs are associated with obesity and related metabolic disturbances in adults as well as in children (see [16, 32] for review).

The primary aim of our study was to identify circulating miRNAs potentially associated to early obesity via an integrated study comprising miRNA signatures and bioinformatic analyses in children participating to the I.Family survey. Here, we present the results of a pilot study on a relatively small sample of children belonging to the Italian cohort of the I.Family survey.

## Results and discussion

### Distinct miRNA expression in plasma of OW/Ob and NW children

**Fig. 1:**
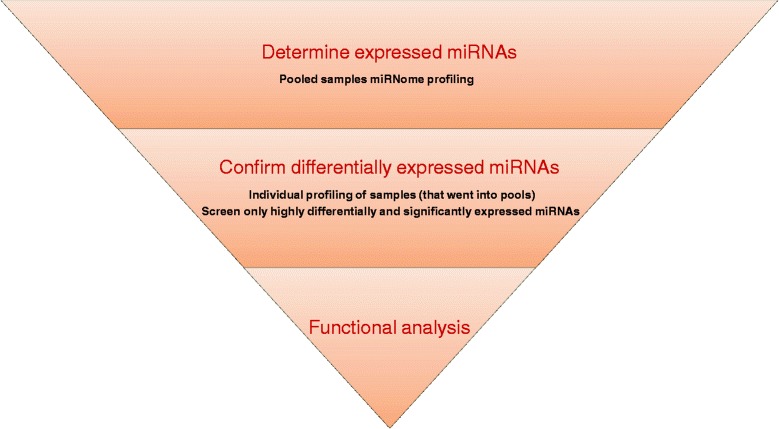
Experimental scheme

The present study was aimed to identify new insights into the pathogenesis of childhood obesity according to the experimental scheme reported in Fig. 1. The main characteristics of the participants are described in Table 1. As time saving and efficiency strategy, samples from ten overweight or obese (OW/Ob) and ten normal weight (NW) were analyzed as pools in triplicate by PCR arrays.

**Table 1:** Anthropometric and metabolic variables of the individuals included in this study

	Normal weight	Overweight/Obese	*p*
Sex (M/F)	5/5	6/4	
Age (years)	10.50 ± 2.76	10.70 ± 1.57	n.s
BMI (kg/cm^2^)	16.45 ± 1.71	31.68 ± 4.32	<0.001
Trg (mg/dL)	53.80 ± 22.08	84.20 ± 30.01	0.02
TC (mg/dL)	146.80 ± 24.10	163.40 ± 33.87	n.s
HDL (mg/dL)	62.30 ± 12.33	52.10 ± 12.37	n.s
LDL (mg/dL)	77.60 ± 22.51	100.70 ± 30.57	n.s
Glu (mg/dL)	91 ± 6.16	94.6 ± 3.53	n.s

Value are mean ± standard deviation

n.s = not significant

The geometric mean Ct value of multiple selected housekeeping genes was 26.13 for NW and 26.38 for OW/Ob. Reverse transcription control average *C*_*t*_ (RTC) was 20.68 for NW and 20.75 for OW/Ob. Absent calls were 1.82 % for NW and 2.34 % for OW/Ob.

**Fig. 2:**
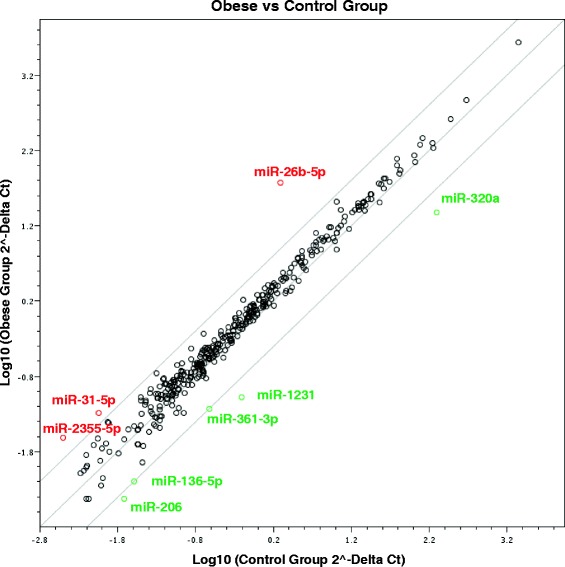
Differential expression of circulating miRNAs in plasma. Comparisons of OW/Ob vs. NW circulating miRNA profiles. The expression levels were queried by qPCR arrays. Means of each data point are presented as a scatter plot (*n* = 3 in each group). Selection threshold is ±4.0. Upregulated miRNAs are marked *red* whereas downregulated miRNAs are marked *green*

Using a significance threshold of *p* < 0.05 and a fold-change threshold of ±4.0, we identified in the pooled samples eight out of 372 total screened miRNAs that differed between the OW/Ob and NW groups (Table 2). A scatter plot (Fig. 2) shows the relative expression levels of miRNAs in OW/Ob vs. NW pooled plasma samples.

**Table 2:** Differential miRNA expression profile determined by gene-arrays screening

miRNA	Fold regulation	
miR-26b-5p	25.3723	↑
miR-31-5p	4.9499	↑
miR-2355-5p	6.5216	↑
miR-1231	−8.7217	↓
miR-361-3p	−4.8918	↓
miR-136-5p	−4.8356	↓
miR-320a	−9.9692	↓
miR-206	−6.0515	↓

miRNAs showing significant differences in expression levels between the compared groups are reported. Analyses were generated setting the threshold at ±4 and *p* value <0.05

up arrow = upregulated; down arrow = downregulated

The eight miRNAs found to be differentially expressed in the pooled analysis were subsequently validated in real-time PCR (RT-qPCR) as distinct assays performed in triplicate in the 20 individual plasma samples. PCR reference gene included the endogenous spike-in *C.el*-miR-39-3p. As reported in Table 3, the differential expression of three miRNAs was statistically confirmed (*p* value <0.05): a twofold upregulation of miR-31-5p, a threefold upregulation of miR-2355-5p, and a 0.5-fold downregulation of miR-206 were observed in OW/Ob as compared with NW.

**Table 3:** Differential miRNA profile confirmed by RT-qPCR

miRNA	OW/Ob	NW	Fold regulation	*p*	
miR-31-5p	3.60 (3.42–3.97)	1.86 (0.47–2.40)	1.92	0.0003	↑
miR-2355-5p	3.65 (2.52–5.35)	1.23 (0.21–1.78)	2.93	0.0166	↑
miR-206	1.28 (0.63–1.83)	2.45 (1.29–4.92)	0.52	0.0461	↓

Differential miRNAs were confirmed by RT-qPCR. Reported values are mean (minimum-maximum). Fold regulation was expressed as fold change with respect to non-obese controls. Reference included the endogenous spike-in *C.el*-miR-39-3p

up arrow = upregulated; down arrow = downregulated

### miRNAs target prediction, KEGG pathway analysis

The specialized algorithm miRPath v2.0 [33] was used to understand the molecular functions of deregulated miRNAs and to predict their putative mRNA targets. To recognize biologically relevant molecular networks and pathways, we further explored miRNA targets by analyzing molecular interactions on the comprehensive knowledge base including the Kyoto Encyclopedia of Genes and Genomes (KEGG) [34] and Ingenuity Pathways Analysis. Both IPA and KEGG technologies provided hints for the functions of miRNAs.

Top pathways predicted to be actively regulated by miRNAs were classified according to the KEGG functional annotation (Table 4).

**Table 4:** MiRPath analysis

KEGG pathway	*p* value
Gap junction	1.13E-13
Arrhythmogenic right ventricular cardiomyopathy (ARVC)	2.90E-05
Notch signaling pathway	0.0003
Regulation of actin cytoskeleton	0.0005
Dorso-ventral axis formation	0.0008
Bacterial invasion of epithelial cells	0.0009
Axon guidance	0.0026
Shigellosis	0.0200
Endocrine and other factor-regulated calcium reabsorption	0.0397
Glycosaminoglycan biosynthesis—heparan sulfate/heparin	0.0483
Pathways in cancer	0.0490
Thyroid cancer	0.0500

Target genes were classified according to KEGG functional annotations to identify top pathways that were actively regulated by miRNAs. Merged *p* value is extracted by combining calculated significance levels using Fisher’s meta-analysis method

Results of mir-31-5p MiRPath analysis on experimentally validated miRNA interactions (derived from DIANA-TarBase v6.0) or predicted targets (provided by the DIANA-microT-CDS algorithm) are reported in Table 5.

**Table 5:** miRPath analysis of Hsa-mir-31-5p interactions

(A) KEGG-predicted pathway derived from DIANA-microT-CDS database	*p* value	genes
Metabolism of xenobiotics by cytochrome P450	1.46E-07	3
Inositol phosphate metabolism	0.00442	4
Fatty acid metabolism	0.02773	2
(B) KEGG-predicted targets provided by the DIANA-TarBase v6.0 database	*p* value	genes
Regulation of actin cytoskeleton	6.24E-06	5
Bacterial invasion of epithelial cells	1.79E-05	3
Axon guidance	8.85E-05	3
Shigellosis	0.00062	2
Pathogenic *Escherichia coli* infection	0.00276	2
Chemokine signaling pathway	0.00653	3
Pertussis	0.00746	2
Hypertrophic cardiomyopathy (HCM)	0.00979	2
Dilated cardiomyopathy	0.01157	2
T cell receptor signaling pathway	0.01604	2
Leukocyte transendothelial migration	0.01604	2
Arrhythmogenic right ventricular cardiomyopathy (ARVC)	0.01604	2
Dorso-ventral axis formation	0.03214	1

miRPath analysis of miR-31-5p (A) of predicted targets provided by the DIANA-microT-CDS algorithm or (B) of experimentally validated miRNA interactions derived from the DIANA-TarBase v6.0. Target pathways were classified according to KEGG functional annotations

Computational prediction of miR-31 identified the following targets in the “fatty acid degradation” pathway: (a) the enoyl-CoA hydratase, a key enzyme in the breakdown of fatty acids essential to metabolizing fatty acids to produce both acetyl CoA and energy, and (b) the alcohol dehydrogenase 1A (Additional file 1: Figure S1).

Among the top predicted targets of miR-31-5p, we also identify the CCAAT-enhancer-binding protein-α (C/EBPα), a main modulator of the expression of genes involved in cell cycle regulation as well as adipocyte functions during adipogenesis (Fig. 2).

As relevant miR-206 targets, we recognized the “insulin signaling,” the “pentose phosphate” pathways, and the liver X receptor-α (LXRα), a ligand-dependent transcription factor playing a relevant role in the metabolism and homeostasis of lipids, cholesterol, bile acids, and steroid hormones with a preeminent expression in the liver.

Further, we identified the oleoyl-ACP hydrolase, a medium-chain acyl-[acyl-carrier-protein] hydrolase in the “fatty acid biosynthesis” pathway as a target of miR-2355 (*p* = 0.005).

Finally, the target prediction of concurrently deregulated miRNAs gave evidence for the “Notch” signaling (*p* = 0.0008) (Additional file 2: Figure S2) and the “Arrhythmogenic right ventricular cardiomyopathy” pathways (*p* = 2.905898 × 10^−05^) (Additional file 3: Figure S3).

## Discussion

We identified three circulating miRNAs differentially expressed in plasma samples of OW/Ob children. Bioinformatic exploratory analysis predicted their involvement in critical pathways including lipid metabolism and adipocyte differentiation.

Adipose tissue is involved in pathophysiological processes because it influences metabolism. In adipogenesis, pluripotent adipose-derived mesenchymal stem cells differentiate into adipocytes. This process is finely regulated by both hormones and transcription factors in a complex signaling network. A number of miRNAs and their targets were recently identified to play regulatory roles in adipocyte differentiation [35–37]. To date, up to 221 miRNAs have been found to be expressed or dysregulated in adipose tissue or adipocytes in mammals [16].

**Fig. 3:**
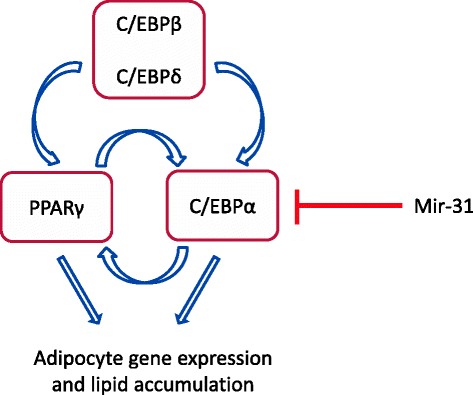
C/EBPα, a key target. The C/EBPα intronless gene encodes a transcription factor containing a basic leucine zipper domain. The protein acts in homodimers or heterodimers that recognize the CCAAT motifs in the promoters of target genes. Complexes modulate the expression of genes involved in cell cycle regulation as well as adipocyte functions: during adipogenesis highly induced genes are synergistically controlled by both PPARγ and C/EBPα

Differentiation from pre-adipocytes into mature adipocytes is orchestrated by several transcription factors such as peroxisome proliferator-activated receptor-γ (PPARγ) and C/EBPs (C/EBPs). C/EBPβ and C/EBPδ are induced by adipogenic stimuli and represent primary regulators of adipogenesis. Targets of C/EBPβ and C/EBPδ are the promoters of the genes encoding crucial adipogenic factors as C/EBPα and PPARγ as well as the sterol regulatory element-binding protein (SREBP1), the key regulator of lipogenic genes. The protein encoded by the C/EBPα intronless gene is a leucine zipper transcription factor which can bind to specific promoters and enhancers as a homodimer or it can form heterodimers with the related proteins C/EBPβ and C/EBPγ (Fig. 3). The C/EBPα has been shown to bind to the promoter and to also modify the expression of the leptin gene that plays a central role in body weight homeostasis. C/EBPα is sufficient to trigger differentiation of pre-adipocytes into mature adipocytes [38]. Peculiarly, PPARγ directly triggers endogenous C/EBPα transcription. In turn, C/EBPα activates the PPARγ gene through a positive feedback loop and thereby promotes adipogenesis [12]. Concurrently, PPARγ and C/EBPα induce the expression of genes that are involved in insulin sensitivity, lipogenesis, and lipolysis and, ultimately, in terminal differentiation and mature functions of adipocytes.

miR-31 directly targets C/EBPα transcript whose levels are in vivo downregulated by miR-31 [37]. Notably, miR-31 expression is inversely correlated with adipogenic differentiation status [39], and consequently, it has a decisive anti-adipogenic function.

We found that miR-31 was significantly upregulated in plasma of OW/Ob children identifying C/EBPα as a privileged target of miR-31. miR-31 upregulation may be considered as an apparent contradiction, but it probably reflects the well-established role of circulating miRNAs as “endocrine signals.” The concept that miRNAs may be transferred from donor to recipient cells is now well established with the relevant implication that miRNAs could affect gene expression distally, so acting as paracrine or endocrine signaling molecules. Accordingly, a panel of “endocrine miRNAs” released from fat cells that may be used as a marker of disturbed adipose tissue function has been characterized [16]. The idea that miR-31 acts as an obesity controlling signal seems to be supported by the literature. Actually, in analyzing the expression of miRNAs in the livers of leptin-deficient obese (ob/ob) mice compared to normal C57BL/6 mice, Li et al. found that ob/ob mice exhibited an upregulation of miR-31 [40], despite its well-established anti-adipogenic role.

Computational prediction of miR-31 recognized several targets in the “fatty acid degradation” pathway (*p* = 0.027) (Additional file 1: Figure S1). Whether miR-31 deregulation plays a causative role or is merely an epiphenomenon in the etiology of obesity still remains to be determined.

In this study, we also observed that miR-206 was significantly downregulated in plasma of OW/Ob children. miR-206 belongs to the cluster of the so-called myomiRs and represents one of the most studied tissue-specific miRNAs involved in skeletal muscle differentiation. Several studies indicate that miR-206 plays a key role in the growth and development of skeletal muscle, promoting myogenic differentiation [41]. Moreover, it has been well established that it is related to the pathogenesis of numerous diseases including heart failure, chronic obstructive pulmonary disease, and Alzheimer’s disease and in numerous kinds of cancers. Interestingly, in most of these diseases, miR-206 levels are downregulated, which leads to the recognition of miRNA as a “disease-avoiding” molecule. Moreover, miR-206 expression is abundant in brown adipocytes but missing in white adipocytes [42]. Remarkably, it has been shown that miR-206 suppresses liver LXRα, a gene target of PPAR, consequently inhibiting lipogenesis and controlling lipid metabolism [43]. The insulin signaling pathway (*p* = 0.01, 11 target genes) and the pentose phosphate pathway (*p* = 0.01, 4 target genes) were also identified as candidate targets of miR-206. Therefore, the reduced level of circulating miR-206 in OW/Ob children may be either representative of a functional decreased synthesis [42] or an epiphenomenon reflecting the reduced relative skeletal muscle mass in the OW/Ob group.

Among the top pathways that were predicted to be targeted by the three differentially expressed miRNAs, “Notch” signaling appears relevant (*p* = 0.0008, 5 target genes, Additional file 2: Figure S2). Notch signaling represents a basic biological pathway primarily related to cell communication and development with extensive integration and crosstalk with other signaling pathways. Perturbations in this pathway have been linked to many genetic disorders and cancers. Accordingly, the direct involvement of Notch signaling has recently been demonstrated in obesity and type 2 diabetes [44]: through transcriptional binding partners FoxO1 and Rbpj, Notch activation enhances pathologic glucose output and fatty liver in obese, in combination with a reduced Akt activation by insulin. Activation of the Notch pathway in obesity results in heightened diabetes and fatty liver production, as a consequence of an augmented lipogenesis that is not blocked despite the reduced insulin signaling. Notably, blocking Notch signaling in mice caused white fat cells to transform into beige fat consequently reducing the risk of obesity and related health problems [44].

While we recognize the limitations and the relative reliability of our target prediction analyses, this hypothesis-generating exercise suggested the involvement of the detected miRNAs in metabolic relevant pathways, to be obviously confirmed by larger and ad hoc designed studies.

Our results confirm those of recent papers indicating that circulating miRNAs are deregulated in obese children [45] and are associated with obesity and lipid abnormalities in adolescents [46]. However, differentially detected miRNA signals in refs. 46 and 47 are not consistent with those identified in our study. Of note, all these miRNAs are present in the 372 miRNA arrays we used in our study but did not pass the threshold we set to consider them as differentially expressed. A solid explanation of these discrepancies is not at hand. Considering the rapidly changing scenario in this field, the interpretation and discussion of the inconsistent findings regarding the association of circulating miRNAs with obesity in children [45, 46] is far beyond the objective of this study. However, recent reviews extensively summarized potentiality, but also uncertainties, of the role of extracellular miRNAs as potential regulators and/or biomarkers of obesity and metabolic diseases [16, 32]. In particular, the inconsistencies of the findings may be ascribed to inter-population differences, but even, and perhaps most likely, to methodological differences in sampling, RNA isolation, detection, and normalization [32]. The need for consistent analysis strategies and quality control has been recently discussed [47].

## Conclusions

This pilot study showed that three circulating miRNAs are differentially regulated in OW/Ob as compared with NW children. Although causal pathways cannot be firmly inferred—but only suggested—by these preliminary results, that deserve confirmation in other populations, it is conceivable that circulating miRNAs may be novel biomarkers of obesity and related metabolic disturbances.

The planned completion of the miRNA studies in the much larger sample of children from the eight European countries participating to the I.Family project will certainly contribute to the ongoing debate whether circulating miRNAs play a role in disease pathogenesis, are (early) indicators of a metabolic dysfunction, or both.

## Methods

### Study cohort

The I.Family project aimed to assess the determinants of eating behavior in children and adolescents of eight European countries and related health outcomes was built on the IDEFICS cohort (http://www.ideficsstudy.eu), established in 2006. We selected our sample among OW/Ob and NW participants of the Italian cohort of the project. The weight status of children was defined according to age- and sex-specific BMI categories [48]. Ten OW/Ob (four females and six males, age 10.7 ± 1.5 years, BMI 31.7 ± 4.3 kg/m^2^) and ten NW (five females and five males, age 10.5 ± 2.7 years, BMI 16.4 ± 1.7 kg/m^2^) children were included in the study.

The study protocol was approved by the local Ethics Committee of the local Health Authority (ASL Avellino) and informed written parental consent was obtained for each participant.

### Sample processing

Whole blood was collected according to the standard operating procedures in BD Vacutainer® blood collection tubes. The optimal approach for miRNA purification from whole blood necessitates collecting fresh blood and processes the sample as quickly as possible. Accordingly, plasma was isolated by centrifugation at 1900×*g* for 10 min at 4 °C in an Eppendorf benchtop centrifuge (Eppendorf, Germany), aliquoted into 1.5 mL Eppendorf tubes, and promptly stored at −80 °C, in the absence of freeze-thaw cycles, until processing.

### miRNA extraction, reverse transcription, and pre-screening

Prior to miRNA extraction, spectrophotometry was carried out on plasma samples to test for hemolysis by measuring the absorbance of free hemoglobin at 414 nm; samples with OD_414_ greater than 0.2 were excluded from the study. RNA was extracted from both plasma pooled samples and from individual plasma samples using the commercial column-based system miRNeasy serum/plasma Kit (Qiagen, Germany) according to the manufacturer’s instructions with minor modifications. Plasma was thawed on ice and spun for 10 min at 13,000×*g* at 4 °C in an Eppendorf benchtop centrifuge (Eppendorf, Germany) to pellet any debris. An aliquot of 200 μL of plasma was transferred to a new microcentrifuge tube and 800 μL of a Qiazol (Qiagen) mixture, containing 1.25 μg/mL of MS2 bacteriophage RNA (Roche Applied Science), was added to the sample. *Caenorhabditis elegans* miR-39 (*C.el*-miR-39) was spiked into the sample (5.6 × 10^8^ molecules) before the extraction process to assess RNA recovery. A rinse step (500 μL Qiagen RPE buffer) was repeated two times. Total RNA was eluted by adding 14 μL of RNase-free water to the membrane of the column and incubating for 1 min before centrifugation at 13,000×*g* for 1 min at room temperature. The obtained RNA was stored at −80 °C.

Complementary DNA (cDNA) was generated by means of the miScript RTII kit (Qiagen), in HiSpec Buffer with miRNA-specific stem-looped RT primers. Nine microliters of isolated RNA were reverse transcribed in 20-μL reactions according to the manufacturer’s recommendations. The obtained cDNA was diluted 1:5.5 in RNase-free water. This procedure was repeated three times. To identify hemolyzed samples, the levels of miR-451, highly abundant in RBCs, were preliminarily assessed in cDNA samples by qPCR. Hemolyzed samples were excluded from the analysis.

### Circulating miRNA profiling

Selected samples from ten OW/Ob and ten NW children (Table 1) were pooled, and miRNAs were extracted as described above. miRNA profiling was performed by using the Serum and Plasma 384HC miScript miRNA PCR Arrays (SABiosciences, Qiagen) to assess the expression of 372 miRNAs typically detectable in serum and plasma. The SYBR green-based qPCR assay was performed with cDNA (dil. 1:225) in 10-uL reaction volume using a 7900HT fast Real-Time PCR System instrument (Applied Biosystems). Reaction conditions were as follows: 15 min at 95 °C and 40 cycles of 15 s at 94 °C, 30 s at 60 °C, and 30 s at 72 °C with a set of technical controls on each plate which included the spike-in control and small non-coding RNAs (SNORDs) as reference genes. References for data normalization included the spike-in *C.el*-miR-39-3p, SNORD61, SNORD68, SNORD72, SNORD95, and SNORD96A, which have been verified to hold relatively stable expression levels across tissues and cell types. Normalization of expression was done using the geometric mean of the endogenous controls. All assays were inspected for distinct melting curves, and the Tm was checked. The amplification efficiency was also calculated. Ct values >35 were considered as negative amplification. Data that did not pass these criteria were omitted from further analysis. The efficiency of reverse transcription was assessed with miRTC.

Statistically significant deregulated miRNAs with a fold-change ≥4.0 were selected and further validated by individual microRNA assays (SABiosciences, Qiagen) performed on the individual plasma samples of ten OW/Ob and ten NW children, using the miScript SYBR green PCR kit (Qiagen) according to the manufacturer’s instructions. RT-qPCR was performed in triplicates using the following conditions: 95 °C for 15 min, followed by 40 cycles of 94 °C for 15 s, 55 °C for 30 s, and 70 °C for 30 s. Relative miRNA levels were determined by the ^ΔΔ^Ct method using the *C.el*-miR-39 as endogenous normalizer.

### Data analysis

On pooled samples, RT-qPCR quantifications were performed by using SDS 2.3 (Life Technologies, USA) to generate Ct values.

Gene Arrays data were organized in Excel (Microsoft), and the integrated web-based software package for miScript Arrays analysis was exploited. This software performs quantification using the ^ΔΔ^Ct method and interpretation of the control assays. Tools are available at the SABiosciences data analysis web portal.

Data on the individual samples are presented as the average of at least three independent experiments ± s.e.. Data Assist 3.1 software packages (Life Technologies, USA) were used to generate relative “fold-change” values. Student’s *t* test, performed with the GraphPad Prism 6 software, was used to determine the significance of any difference in the levels of miRNA expression between NW and OW/Ob subjects with alpha <0.05.

Obesity-associated miRNAs were tested using the well-established target prediction tool miRPath v2.0 [33]. miRPath achieves advanced analysis pipelines, such as hierarchical clustering of miRNAs and pathways based on the levels of their interactions. miRNA targets (in CDS or 3′-UTR regions) were predicted by the DIANA-microT-CDS algorithm or even experimentally validated miRNA interactions derived from DIANA-TarBase v6.0. Predicted and/or validated interactions were subsequently combined with merging and meta-analysis algorithms.

All the predicted targets were further analyzed through the use of Kyoto Encyclopedia of Genes and Genomes (KEGG) [34] and QIAGEN’s Ingenuity® Pathway Analysis (IPA®, QIAGEN Redwood City, www.qiagen.com/ingenuity). IPA identified lists of genes that satisfy specific biological criteria scoring these genes towards pathways in the Ingenuity Knowledge Base which embraces a large database of biological and chemical relationships extracted from scientific literature.

## Additional files

Additional file 1: Figure S1. In the highly predicted pathway ‘Fatty acid metabolism’ [hsa00071] targets of miR-31-5p are highlighted. Relevant pathways predicted to be targeted by the differentially expressed miRNAs. (PPTX 64 kb) Additional file 2: Figure S2. miRNA targets are highlighted in the NOTCH signaling pathway. (PPTX 42 kb) Additional file 3: Figure S3. Targets actively regulated by miRNAs in “Arrhythmogenic right ventricular cardiomyopathy” pathway. (PPTX 301 kb)
