# Supplementary material for: Circulating microRNAs are deregulated in overweight/obese children: preliminary results of the I.Family study
Source: Genes Nutr. 2016 Mar 21;11:7. doi: 10.1186/s12263-016-0525-3 (PMC4968450; doi:10.1186/s12263-016-0525-3)
Supplement: Additional file 1: Figure S1. — In the highly predicted pathway ‘Fatty acid metabolism’ [hsa00071] targets of miR-31-5p are highlighted. Relevant pathways predicted to be targeted by the differentially expressed miRNAs. (PPTX 64 kb) [file 12263_2016_525_MOESM1_ESM.pptx]

## Slide 1
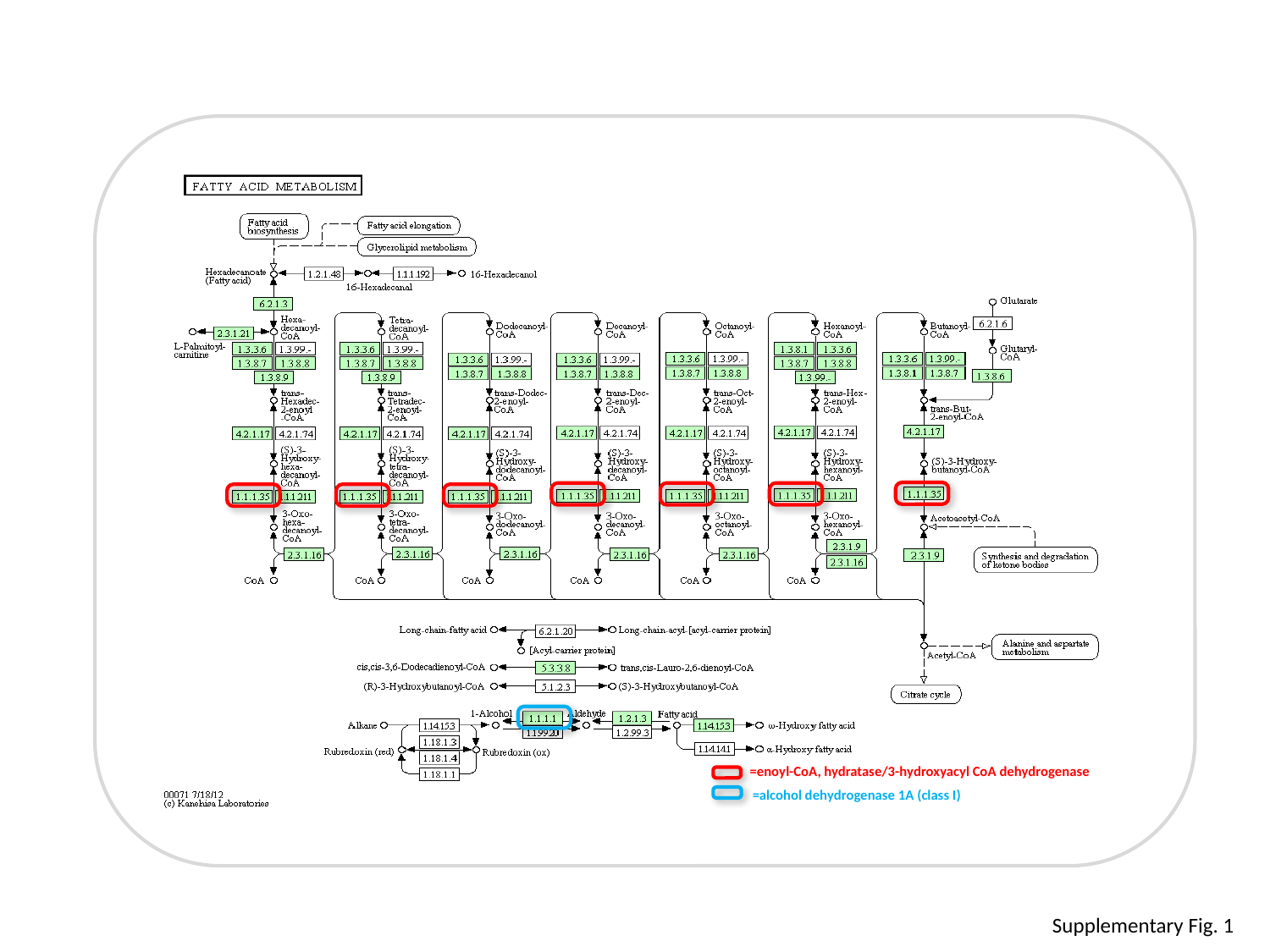

=enoyl-CoA, hydratase/3-hydroxyacyl CoA dehydrogenase
=alcohol dehydrogenase 1A (class I)
Supplementary Fig. 1
