# Supplementary figures and images for: Circulating microRNAs are deregulated in overweight/obese children: preliminary results of the I.Family study
Source: Genes Nutr. 2016 Mar 21;11:7. doi: 10.1186/s12263-016-0525-3 (PMC4968450; doi:10.1186/s12263-016-0525-3)

## Slide 1
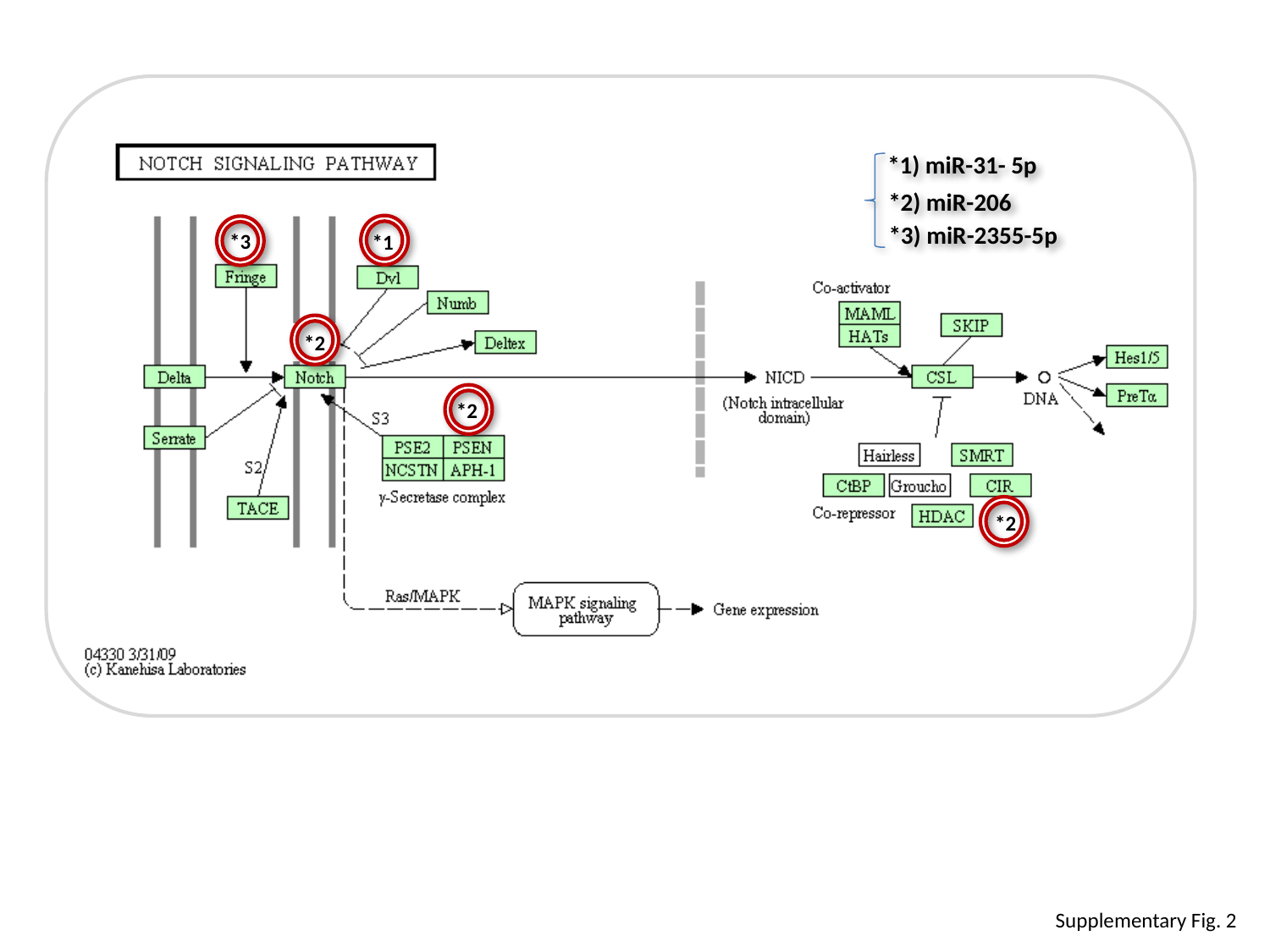

*1) miR-31- 5p
*2) miR-206
*3) miR-2355-5p
*3
*1
*2
*2
*2
Supplementary Fig. 2

Supplement: Additional file 2: Figure S2. — miRNA targets are highlighted in the NOTCH signaling pathway. (PPTX 42 kb) [file 12263_2016_525_MOESM2_ESM.pptx]

## Slide 1
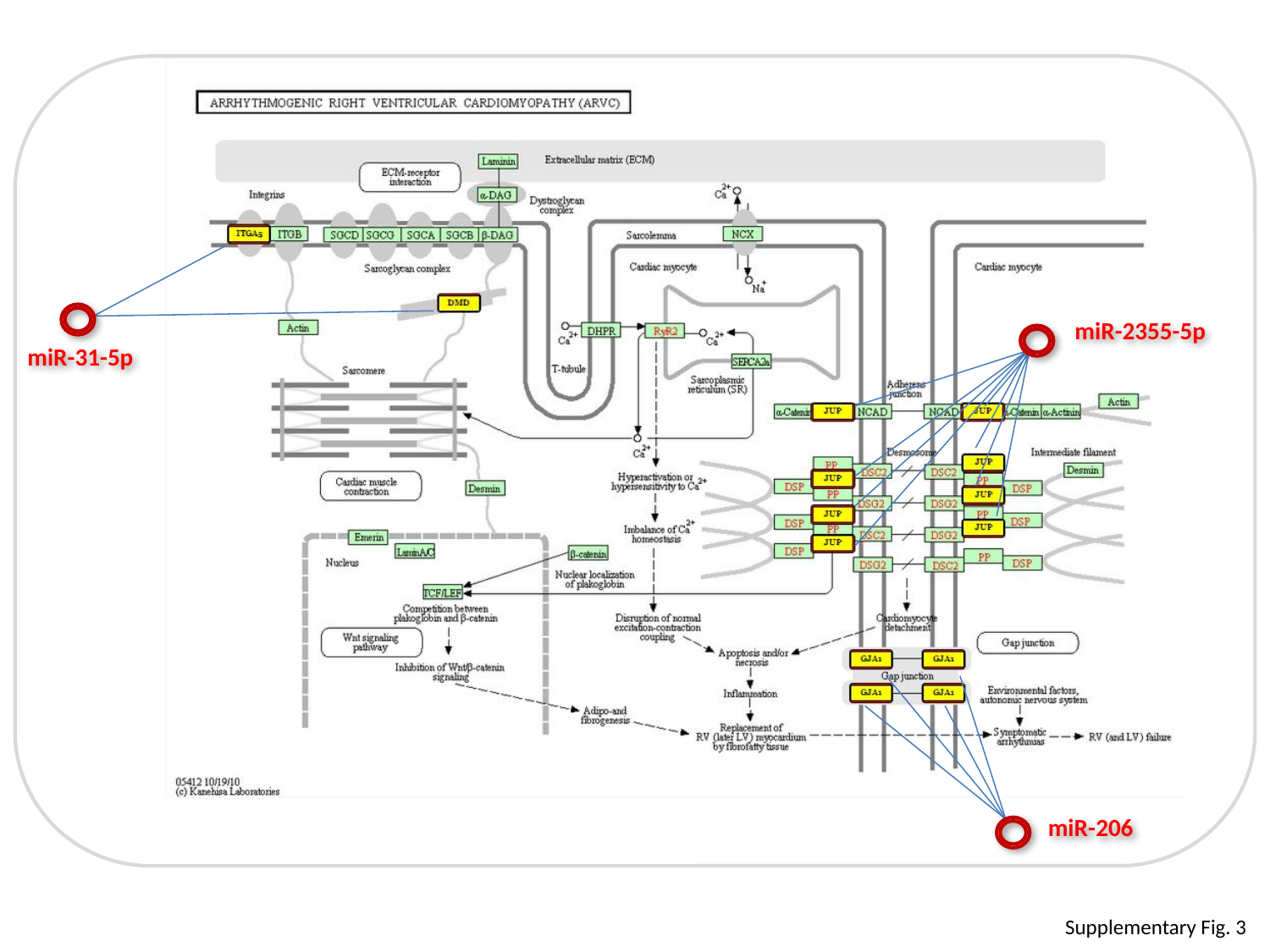

miR-2355-5p
miR-31-5p
miR-206
Supplementary Fig. 3

Supplement: Additional file 3: Figure S3. — Targets actively regulated by miRNAs in “Arrhythmogenic right ventricular cardiomyopathy” pathway. (PPTX 301 kb) [file 12263_2016_525_MOESM3_ESM.pptx]
